# Epstein-Barr Virus Promotes the Production of Inflammatory Cytokines in Gingival Fibroblasts and RANKL-Induced Osteoclast Differentiation in RAW264.7 Cells

**DOI:** 10.3390/ijms23020809

**Published:** 2022-01-12

**Authors:** Sho Yokoe, Akira Hasuike, Norihisa Watanabe, Hideki Tanaka, Hiroyuki Karahashi, Shin Wakuda, Osamu Takeichi, Takayuki Kawato, Hideki Takai, Yorimasa Ogata, Shuichi Sato, Kenichi Imai

**Affiliations:** 1 Department of Periodontology, Nihon University School of Dentistry, Tokyo 101-8310, Japan; desh18040@g.nihon-u.ac.jp (S.Y.); hasuike.akira@nihon-u.ac.jp (A.H.); watanabe.norihisa@nihon-u.ac.jp (N.W.); deyu20009@g.nihon-u.ac.jp (H.K.); desh21024@g.nihon-u.ac.jp (S.W.); satou.shuuichi@nihon-u.ac.jp (S.S.); 2 Department of Microbiology, Nihon University School of Dentistry, Tokyo 101-8310, Japan; 3 Department of Oral Health Sciences, Nihon University School of Dentistry, Tokyo 101-8310, Japan; tanaka.hideki@nihon-u.ac.jp (H.T.); kawato.takayuki@nihon-u.ac.jp (T.K.); 4 Department of Endodontics, Nihon University School of Dentistry, Tokyo 101-8310, Japan; takeichi.osamu@nihon-u.ac.jp; 5 Department of Periodontology, Nihon University School of Dentistry at Matsudo, Chiba 271-8587, Japan; takai.hideki@nihon-u.ac.jp (H.T.); ogata.yorimasa@nihon-u.ac.jp (Y.O.)

**Keywords:** periodontitis, Epstein–Barr virus, proinflammatory cytokines, osteoclast, gingival fibroblasts

## Abstract

Periodontitis is an inflammatory condition that causes the destruction of the supporting tissues of teeth and is a major public health problem affecting more than half of the adult population worldwide. Recently, members of the herpes virus family, such as the Epstein–Barr virus (EBV), have been suggested to be involved in the etiology of periodontitis because bacterial activity alone does not adequately explain the clinical characteristics of periodontitis. However, the role of EBV in the etiology of periodontitis is unknown. This study aimed to examine the effect of inactivated EBV on the expression of inflammatory cytokines in human gingival fibroblasts (HGFs) and the induction of osteoclast differentiation. We found that extremely high levels of interleukin (IL)-6 and IL-8 were induced by inactivated EBV in a copy-dependent manner in HGFs. The levels of IL-6 and IL-8 in HGFs were higher when the cells were treated with EBV than when treated with lipopolysaccharide and lipoteichoic acid. EBV induced IκBα degradation, NF-κB transcription, and RAW264.7 cell differentiation into osteoclast-like cells. These findings suggest that even without infecting the cells, EBV contributes to inflammatory cytokine production and osteoclast differentiation by contact with oral cells or macrophage lineage, resulting in periodontitis onset and progression.

## 1. Introduction

Periodontitis, which used to be termed chronic periodontitis, is a chronic inflammatory disease that causes the destruction of periodontal tissues and alveolar bone; it is among the most prevalent infectious diseases in the world. Although no single etiological agent has been identified, a number of bacteria, such as *Porphyromonas gingivalis*, are believed to be associated with the disease and are used as diagnostic markers [1]. However, bacterial activity alone cannot explain the several clinical characteristics of periodontitis [2]. Several reports have demonstrated the absence of periodontal bacteria in patients with periodontitis, and no significant difference has been found in the prevalence of bacteria between healthy and diseased periodontium [3,4,5]. Recently, a positive association has been reported between periodontitis and infection with herpesviruses, such as the Epstein–Barr virus (EBV) and human cytomegalovirus (CMV) [2,6]. EBV is an enveloped herpes virus with double-stranded DNA and only infects humans. During a primary EBV infection, the virus undergoes lytic replication in epithelial cells and B cells, in which it establishes latency [7]. Periodically, the virus can reactivate, and it is commonly found in the saliva of infected persons [2].

Recently, we demonstrated that *P.* *gingivalis* can induce EBV reactivation through epigenetic regulation [8]. Many studies have found a higher prevalence of EBV DNA in the gingival tissue, gingival crevicular fluid, and subgingival plaque of patients with periodontitis [9,10,11,12,13]. We also reported that EBV DNA was more frequently detected in patients with deeper probing depth sites than in those with shallow probing depth sites or healthy controls. The range of counts of EBV DNA in the periodontal pockets of periodontitis patients was 3.74 × 10^3^–2.83 × 10^9^ copies/mL [14,15]. A large number of EBV-encoded small RNA (EBER) positive cells were found in the gingival tissue of patients with periodontal disease [14]. Vincent-Bugnas et al. reported that the level of gingival epithelial EBV infection is correlated with the severity of periodontitis [16]. Treatment with an antiherpesvirus drug decreased the presence of EBV to the detection limit, and the periodontal condition improved dramatically [17]. These observations indicate that EBV is involved in the etiology of periodontal disease. However, despite its importance, the molecular mechanisms by which EBV affects the pathogenesis and progression of periodontal disease remain poorly understood.

It has been demonstrated that periodontopathic bacterial infections and their associated bacterial products, such as lipopolysaccharide (LPS), stimulate the production of inflammatory cytokines such as interleukin (IL)-6 and IL-8 from human gingival fibroblasts (HGFs) and monocytes, leading to connective tissue destruction and bone resorption in periodontal patients [1,18]. Both LPS and inflammatory cytokines promote osteoclastogenesis and enhance osteoclast-mediated bone resorption by promoting the expression of the receptor activator of nuclear factor kappa B (NF-κB) ligand (RANKL) [1,18,19].

EBV can also trigger the cells of the immune system to produce cytokines and monokines, and inflammatory mediators play an important role in the pathogenesis and eventual outcome of EBV infection [7]. Although the main cells targeted by EBV infection are B cells, several studies have reported that UV- or heat-inactivated EBV could stimulate the production of inflammatory cytokines in monocytes and neutrophils [20,21,22,23]. These observations indicate that even when EBV has not infected the cells, simple contact with host cells can induce the production of inflammatory cytokines involved in the progression of EBV infection.

The purpose of this study is to examine whether inactivated EBV affected the expression of inflammatory cytokines in HGFs. HGFs are the major cells in periodontal connective tissues, providing a tissue framework for tooth anchorage and functioning as a regulator of the production of inflammatory molecules. We also investigated the potential of inactivated EBV for inducing osteoclast differentiation.

To the best of our knowledge, this is the first time that inactivated EBV has been shown to markedly induce the production of inflammatory cytokines, the activation of nuclear factor kappa B (NF-κB) in oral cells, and the differentiation of RAW264.7 cells into osteoclast-like cells.

## 2. Results

### 2.1. Effect of EBV on IL-6 and IL-8 mRNA Expression in Gingival Fibroblasts

**Figure 1:**
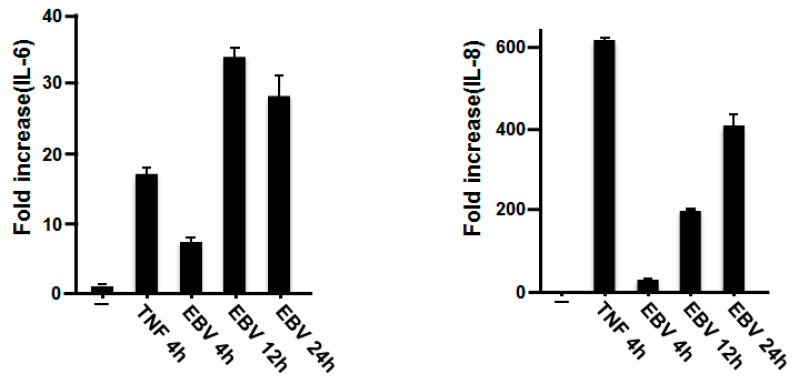
Effects of EBV on the expression of IL-6 and IL-8 mRNA in HGFs. HGFs were treated with inactivated EBV (5000 copies/mL) or TNF-α (1 ng/mL) for the indicated times. “-” indicates treatment with virus-treated stock solution as a control. RT-PCR analysis was performed to detect the expression of IL-6 and IL-8 mRNA with specific primers. IL-6 and IL-8 mRNA levels were normalized to glyceraldehyde-3-phosphate dehydrogenase (GAPDH) mRNA levels, and they are shown as fold increase. EBV, Epstein–Barr virus; IL, interleukin; HGFs, human gingival fibroblasts; TNF-α, tumor necrosis factor-α; RT-PCR, real-time polymerase chain reaction; GAPDH, glyceraldehyde-3-phosphate dehydrogenase.

To investigate the effect of EBV on the expression of IL-6 and IL-8 from HGFs, we performed a real-time polymerase chain reaction (RT-PCR) analysis. Because we detected EBV DNA in deeper probing pocket depths sites (≥5 mm) ranging between 3.74 × 10^3^ and 2.83 × 10^9^ copies/mL in Japanese periodontitis patients [15], 5000 copies/mL of EBV were used to treat HGFs for different periods of time. The extent of stimulation was comparable with that elicited by tumor necrosis factor-α (TNF-α) as a positive control. EBV substantially increased the expression of IL-6 and IL-8 mRNA (Figure 1). The IL-6 mRNA level was upregulated in HGFs 4 h after incubation with inactivated EBV and reached a maximum after 12 h of stimulation. High time-dependent expression of IL-8 mRNA was observed in response to incubation with EBV.

### 2.2. EBV Promoted the Production of IL-6 and IL-8 in HGFs

**Figure 2:**
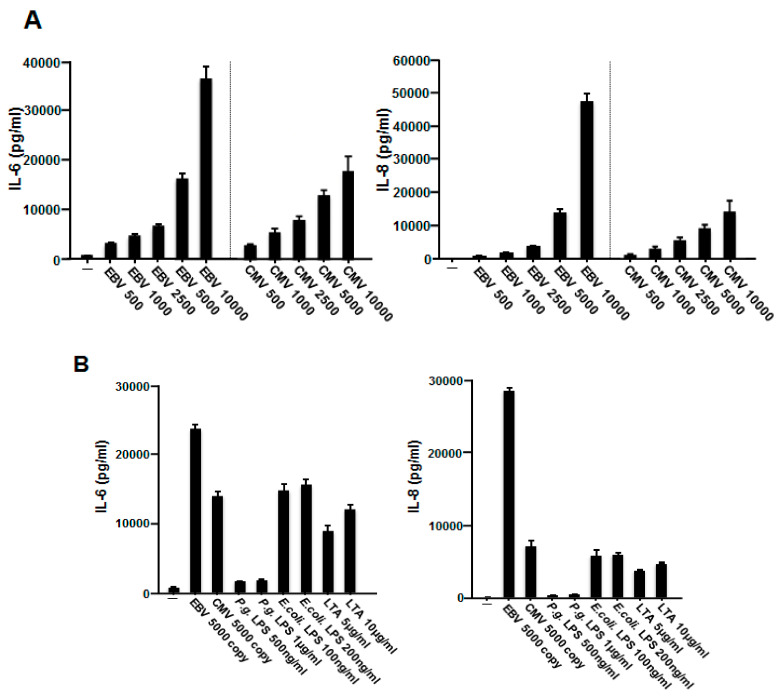
EBV induced the release of IL-6 and IL-8 from HGFs. (**A**) HGFs were treated with inactivated EBV or CMV at the indicated copy numbers (copies/mL) for 48 h. Culture supernatants were collected, and IL-6 and IL-8 levels were measured using ELISA. (**B**) HGFs were treated with inactivated EBV (5000 copies/mL), CMV (5000 copies/mL), *P. gingivalis* LPS (500 ng or 1 μg/mL), *E. coli* LPS (100 or 200 ng/mL), or *S. aureus* LTA (5 or 10 μg/mL) for 48 h, and then, IL-6 and IL-8 levels in the supernatants were detected using ELISA. Experiments were performed in triplicate, and data are presented as mean ± standard deviation (pg/mL). EBV, Epstein–Barr virus; IL, interleukin; HGFs, human gingival fibroblasts; CMV, human cytomegalovirus; LPS, lipopolysaccharide; LTA, lipoteichoic acid (LTA); ELISA, enzyme-linked immunosorbent assay.

We next investigated the effect of EBV on the production of IL-6 and IL-8. HGFs were incubated with or without inactivated EBV for 48 h, and the amounts of both cytokines were measured using an enzyme-linked immunosorbent assay (ELISA). As shown in Figure 2A, the production of both IL-6 and IL-8 was induced by EBV in HGFs in a copy-dependent manner. Although CMV alone could induce the production of IL-6 and IL-8, significantly more inflammatory cytokines were produced by treatment with EBV than by treatment with CMV.

LPS, the major pathogenic component of the cell wall of *P. gingivalis*, can activate host immune responses, including the production of inflammatory cytokines and chemokines, leading to the progression of periodontal disease [1]. LPS from *E. coli* and lipoteichoic acid (LTA) from *S. aureus* were employed as stimulators for the production of inflammatory cytokines in HGFs. To compare the effects of EBV on HGFs, we also tested LPS and LTA. As shown in Figure 2B, the levels of IL-6 and IL-8 in HGFs were highest when the cells were treated with EBV rather than with *P. gingivalis* LPS, *E. coli* LPS, or LTA. These findings suggested that EBV contributes to the progression of periodontal disease by promoting the production of inflammatory cytokines.

### 2.3. NF-κB Was Involved in the Production of IL-6 and IL-8 Induced by Inactivated EBV in HGFs

**Figure 3:**
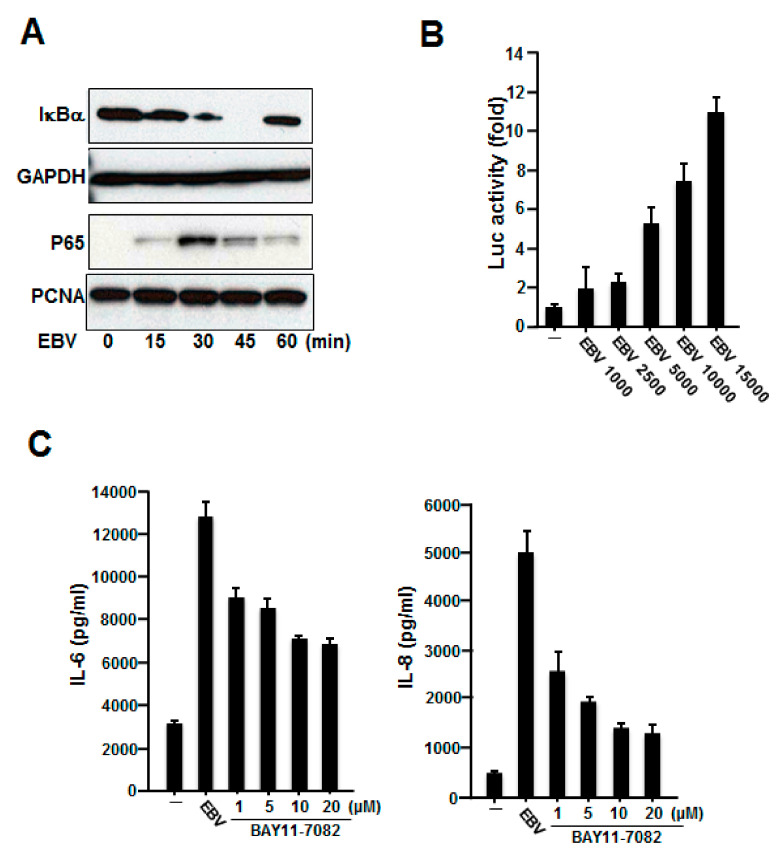
Activation of NF-κB by EBV and its involvement in inactivated EBV-induced IL-6 and IL-8 production. (**A**) Inactivated EBV-induced degradation of IκBα and translocation of p65 to the nucleus. HGFs were treated with inactivated EBV (5000 copies/mL) for the indicated time periods. Cytoplasmic extracts were fractionated using sodium dodecyl sulfate-polyacrylamide gel electrophoresis and immunoblotted with anti-IκBα and GAPDH antibodies. Nuclear extracts were immunoblotted with anti-p65 and PCNA antibodies. (**B**) Activation of NF-κB-dependent gene expression by inactivated EBV. The 4xκB-luc reporter plasmid at 20 ng was transfected into HGFs, which were incubated for 24 h and stimulated with the indicated amount of inactivated EBV for an additional 8 h. The luciferase activity of each cell lysate was then measured. All experiments were performed in triplicate, and the data are presented as the fold increase in luciferase activity (mean ± standard deviation) relative to the control. (**C**) Effects of BAY11-7082 on inactivated EBV-induced IL-6 and IL-8 production. HGFs were pretreated with various concentrations of BAY11-7082 for 1 h and then incubated with inactivated EBV (5000 copies/mL) for an additional 24 h. The cell culture supernatants were analyzed for IL-6 and IL-8 levels using ELISA. NF-κB, nuclear factor kappa B; EBV, Epstein–Barr virus; IL, interleukin; HGFs, human gingival fibroblasts; ELISA, enzyme-linked immunosorbent assay; PCNA, proliferating cell nuclear antigen.

NF-κB is an inducible cellular transcriptional factor that regulates a wide variety of cellular genes involved in the control of the inflammatory and immune responses [24]. NF-κB is normally present in the cytoplasm in association with its inhibitory protein IκB. Upon stimulation, signaling events are initiated, leading to the activation of the IκB kinase (IKK) complex, which promotes the phosphorylation of IκB on two specific serine residues (Ser32/36) in IκBα. This phosphorylation leads to the degradation of IκB by the 26S proteasome and, thus, the nuclear translocation of NF-κB [24]. Because NF-κB is part of one of the most important pathways mediating the generation of IL-6 and IL-8 [24,25], we examined whether inactivated EBV can activate NF-κB, and, if so, whether NF-κB is involved in the expression of these cytokines induced by inactivated EBV. To assess whether EBV can activate NF-κB, gingival fibroblasts were stimulated with inactivated EBV for different times and analyzed for IκBα and NF-κB (p65) expression using western blot analysis. As shown in Figure 3A, degradation of IκBα was observed as early as 15 min after stimulation of these cells by inactivated EBV. We also observed that inactivated EBV stimulated the nuclear translocation of NF-κB p65. To further examine whether NF-κB activation by inactivated EBV in HGFs occurred at the transcriptional level, we employed luciferase assays using a reporter plasmid whose expression is controlled by NF-κB. Figure 3B shows that inactivated EBV stimulated the activation of NF-κB-dependent genes, such as 4xκB-luc, in a copy number-dependent manner. These results suggest that EBV could activate the NF-κB pathway in HGFs.

We further examined whether NF-κB is involved in the induction of the expression of IL-6 and IL-8 in HGFs by inactivated EBV. We used an NF-κB selective inhibitor, BAY11-70582. Gingival fibroblasts were preincubated with various concentrations of BAY11-70582 before stimulation with inactivated EBV, and the amounts of these cytokines were determined (Figure 3C). We found that BAY11-70582 inhibited the production of IL-6 and IL-8 induced by EBV. These results showed that NF-κB signaling contributed to the EBV-induced production of IL-6 and IL-8 in HGFs.

### 2.4. EBV Promoted RANKL-Induced Osteoclast Differentiation

**Figure 4:**
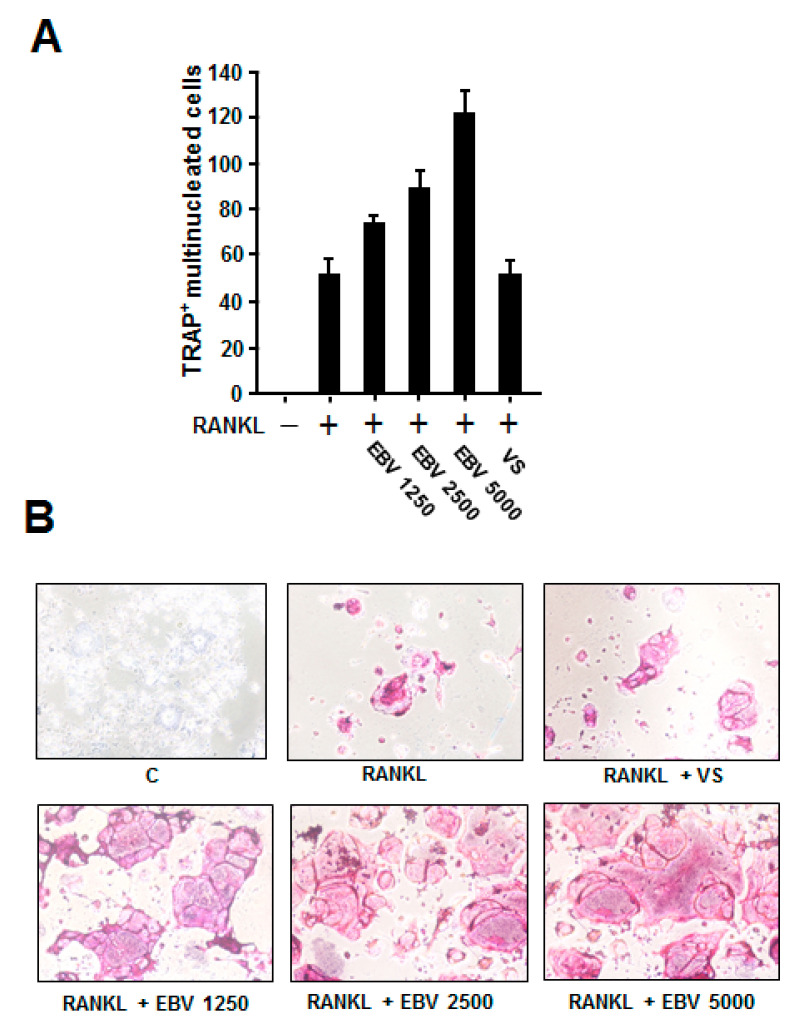
Induction of RANKL-induced osteoclast differentiation by EBV. RAW264.7 cells were seeded in a 96-well plate and treated with recombinant RANKL (50 ng/mL) in the absence or presence of the indicated copy numbers of EBV (copies/mL) for 4 days, followed by TRAP staining. (**A**) The number of TRAP-positive multinucleated cells present in each well was counted. “VS” indicates treatment with virus-treated stock solution. (**B**) TRAP-positive multinucleated cells were observed by light microscopy. RANKL, receptor activator of nuclear factor kappa B ligand; EBV, Epstein–Barr virus; TRAP, tartrate-resistant acid phosphatase.

Osteoclasts originate from the monocyte/macrophage lineage and demineralize and degrade the bone matrix [19]. Osteoclast differentiation is regulated by the binding of RANK present on the surface of preosteoclasts to its ligand RANKL, which leads to the activation of NF-κB [19]. To examine the effect of EBV on RANKL-induced osteoclastogenesis, macrophage-like RAW264.7 cells were treated with different copy numbers of EBV in the presence of recombinant RANKL. Although RANKL stimulation induced the formation of osteoclast-like tartrate-resistant acid phosphatase (TRAP)-positive multinuclear giant cells, interestingly, EBV treatment upregulated their number in a copy number-dependent manner (Figure 4A). Higher magnification images also revealed that EBV increased both the number and size of osteoclast-like cells in a copy number-dependent manner (Figure 4B).

## 3. Discussion

Although there is evidence for an association between EBV and periodontitis, the way in which EBV affects the pathogenesis and progression of periodontitis has not been well understood. It is known that excessive and/or continuous production of inflammatory cytokines occurs in response to the presence of periodontopathic bacteria and their products, such as LPS, in the periodontal tissue of periodontitis patients and is an important determinant for the onset and progression of periodontal disease. To clarify this issue, it is important to identify and characterize any complex proinflammatory cytokine network involved in periodontal pathogenesis and its link to EBV. In the present study, we investigated the effects of inactivated EBV on the expression of inflammatory cytokines in HGFs. We demonstrated that EBV could activate NF-κB and could subsequently promote the production of IL-6 and IL-8 in HGFs.

IL-6 activity is involved in the stimulation of acute-phase protein synthesis, leukocyte recruitment, B-cell differentiation, and T-cell activation in many chronic inflammatory diseases [26]. IL-8, a potent neutrophil chemoattractant and activator, has been associated with the pathogenesis of periodontal diseases and the accumulation and degranulation of neutrophils, with the subsequent destruction of normal tissue [27]. These cytokines could also stimulate bone resorption by promoting the formation of osteoclasts [28]. Many investigators have detected IL-6 and IL-8 in gingival cavity fluid (GCF), and their levels in GCF are closely associated with the severity of inflammatory responses and periodontal tissue destruction [28,29,30,31,32,33]. Reinhardt et al. reported that because IL-6 levels in GCF of periodontitis patients were higher than IL-1 levels, IL-6 plays an important role in the progression of periodontal disease [33]. It has been shown that, primed by IL-1β, IL-8 markedly upregulates neutrophils for elastase release, giving rise to a significant amplification of periodontal inflammation [32]. We observed that the production of IL-6 and IL-8 induced by EBV was significantly stronger than that induced by *P. gingivalis* LPS. Because these inflammatory cytokines are multifunctional and exert their effects in a paracrine and autocrine fashion to modulate the inflammatory and immune responses of HGFs, these findings suggested that EBV contributes to the progression of periodontal disease by promoting the production of inflammatory cytokines.

A number of highly inducible genes encoding cytokines and chemokines contain NF-κB-binding sites in their proximal promoters [24], and activation of NF-κB plays a central role in virus-dependent cytokine expression and pathology. We observed that EBV could stimulate the degradation of IκBα and the phosphorylation of NF-κB p65 and NF-κB-dependent genes, such as 4xκB-luc. Inhibition of NF-κB reduced the production of IL-6 and IL-8 induced by inactivated EBV in HGFs. Although our data suggested that NF-κB was involved in EBV-induced cytokine production in HGFs, EBV also results in the activation of other transcription factors, as well as members of signaling pathways, such as mitogen-activated protein kinases. Thus, further study is needed to understand what kind of signaling pathway is involved in the induction of inflammatory cytokines by EBV in HGFs.

Because inactivated EBV was used for the stimulation of HGFs, our results suggest that infection with EBV is not required for the induction of inflammatory cytokines and NF-κB activation in HGFs. EBV infects B cells and replicates in the epithelial cells of the oropharynx, suggesting that EBV can interact with other cell types, such as T cells and thymocytes [7,16]. EBV can also interact with monocytes and neutrophils and can modulate the expression of inflammatory cytokines, such as IL-1, IL-8, macrophage inflammatory protein-1α, and granulocyte-macrophage colony-stimulating factor, without penetrating these cells [20,21,22,23]. Tanner et al. reported that EBV glycoproteins gp350 and gp220 induced the production of IL-6, so contact between the structural proteins of the virus, such as glycoproteins, and the cells might be necessary for the induction of cytokine expression by inactivated EBV [34]. These observations, including our results, suggest that even when EBV has not infected B cells, it contributes to the onset and progression of periodontitis by simple contact with and subsequent activation of macrophages, fibroblasts, and neutrophils in the vicinity of B cells, resulting in the production and release of inflammatory cytokines. Although CD21 and Toll-like receptors are also expressed in oral cells [18,35], it is necessary to clarify which receptors are involved in EBV-induced inflammatory cytokines in HGFs.

A major clinical symptom of periodontal disease is alveolar bone loss due to excessive resorption by osteoclasts [1,18]. *P. gingivalis* affects osteoclast formation in various ways, such as by interacting directly with osteoclast precursors, including bone marrow macrophages, or indirectly by activating HGFs and osteoblasts, which aid osteoclast formation [18]. LPS is also involved in osteoclast differentiation via inducing RANKL expression [18,19]. In this study, we found that EBV could induce the differentiation of RAW264.7 cells into osteoclast-like cells. Most recently, we also reported that the human immune response to EBV infection may induce human osteoclast activation and cause erosive arthritis in humanized NOD/Shi-*scid/IL-2Rγ*^null^ (hu-NOG) mice [36]. In addition, we observed that EBER and osteoclasts were detected in the periodontium of EBV-infected hu-NOG mice (data not shown). These observations, including the results of this study, suggest that EBV may be involved in the onset and progression of inflammatory bone diseases, such as periodontal disease and rheumatoid arthritis. Although EBV does not infect mice, presenting a challenge for biomedical research, the mechanism by which EBV induces osteoclast differentiation and whether EBV actually promotes alveolar bone resorption in vivo are important issues that need to be addressed in the near future.

We previously demonstrated that *P.* *gingivalis* can induce EBV reactivation through epigenetic regulation [8]. Significantly high levels of butyric acid have been found in the saliva of periodontitis patients, which could efficiently induce the expression of the EBV lytic switch activator *BZLF1* [37]. We assume that the interaction between EBV and periodontopathic bacteria leads to the following negative chain of pathological events in the oral cavity: (1) poor oral hygiene causes the accumulation of butyric acid-producing periodontopathic anaerobic bacteria, such as *P. gingivalis* and *F. nucleatum*; (2) butyric acid induces EBV reactivation; (3) EBV induces the production of high concentrations of inflammatory cytokines in various types of cells and the formation of osteoclasts without infection; (4) progression of gingival inflammation and alveolar bone resorption; (5) periodontitis escalation.

Periodontitis and EBV are spreading worldwide. Although additional basic and clinical studies are needed, reducing the amount of EBV, as well as the number of bacteria, may be valuable for the prevention and treatment of periodontitis.

## 4. Materials and Methods

### 4.1. Cell Culture

HGFs were maintained at 37 °C in Dulbecco’s modified Eagle’s medium (Sigma Aldrich, St. Louis, MO, USA) with 10% heat-inactivated fetal bovine serum (FBS) (Thermo Fisher Scientific Inc., Rockford, IL, USA), penicillin (100 U/mL), and streptomycin (100 μg/mL). The Ethics Committee of Nihon University School of Dentistry at Matsudo approved the study (EC03-041, EC10-040, and EC14-023), and written informed consent was obtained from each patient after a full explanation of the protocol. The mouse macrophage-like cell line RAW264.7 was purchased from Dainippon Pharmaceutical (Osaka, Japan) and maintained in α-minimal essential medium (WAKO, Osaka, Japan) supplemented with 10% FBS and penicillin/streptomycin.

### 4.2. Reagents and Plasmids

Inactivated EBV (strain: B95-8) and CMV (strain: AD-169) were purchased from ZeptoMetrix (Buffalo, NY, USA). These viruses were isolated from cell culture medium or patient plasma and were purified to a high degree. The viral surface proteins were chemically or enzymatically processed while leaving nucleic acids intact. A virus-treated solution (ZeptoMetrix, Buffalo, NY, USA) was added to the cells as a control. Human recombinant TNF-α was purchased from Roche Diagnostics Deutschland GmbH (Mannheim, Germany). *P. gingivalis* LPS was obtained from Invivogen (St. Louis, MO, USA). *Escherichia coli* LPS and *Staphylococcus* *aureus* LTA were purchased from Sigma Aldrich. Antibodies for, IκBα, p65, PCNA, and GAPDH were purchased from Santa Cruz Biotechnology, Inc. (Santa Cruz, CA, USA). BAY11-7082, an inhibitor of IκB-α phosphorylation, was purchased from Sigma Aldrich. The reporter plasmid used, which expresses firefly luciferase under the control of NF-κB (pGL3-κB luc), was described previously [38,39].

### 4.3. Preparation of mRNA and Real-Time Polymerase Chain Reaction

The experimental procedures for RNA purification and RT-PCR were performed as previously described [39]. Briefly, HGFs were washed once with ice-cold phosphate-buffered saline (PBS) and homogenized with QIAshredder (Qiagen, Hilden, Germany), and total RNA was purified using RNeasy Mini Kits (Qiagen). For cDNA synthesis, 1 μg of total RNA was reverse transcribed using RNA PCR kits (PrimeScript; Takara Bio, Shiga, Japan). The resulting cDNA mixture was subjected to RT-PCR analysis using SYBR Premix Ex *Taq* solution (Takara Bio) containing 10 μM sense and antisense primers. The primer sequences for each amplified gene were as follows: IL-6, forward (5′-CCA TAC CAG GTG CCT TTT GT-3′) and reverse (5′-GAG ACT GGG AAC AGC TGA GG-3′); IL-8, forward (5′-CCA TAC CAG GTG CCT TTT GT-3′) and reverse (5′-GAG ACT GGG AAC AGC TGA GG-3′); IL-8, forward (5′-CTT GTC ATT GCC AGC TGT GT-3′) and reverse (5′-TGA CTG TGG AGT TTT GGC TG-3′); and GAPDH, forward (5′-ACC AGC CCC AGC AAG AGC ACA AG-3′) and reverse (5′-TTC AAG GGG TCT ACA TGG CAA CTG-3′). The PCR assays were performed using TP-800 Thermal Cycler Dice Real-Time System (Takara Bio) and were analyzed using the software provided by the device manufacturer. The thermal cycling conditions were 40 cycles at 95 °C for 5 s, 60 °C for 30 s, and 72 °C for 1 min. All RT-PCR experiments were performed in triplicate, and the specificity of each product was verified by melting curve analysis. The calculated gene expression levels were normalized to GAPDH mRNA levels.

### 4.4. Cytokine Measurements

Cytokine concentrations were measured using human ELISA kits for IL-6 and IL-8 (R&D Systems, Minneapolis, MN, USA) in HGF culture supernatant using the experimental procedures recommended by the manufacturer. All experiments were performed in triplicate, and data are presented as mean ± standard deviation.

### 4.5. Preparation of Whole-Cell and Nuclear Extracts

HGFs (2 × 10^5^ cells/mL) were treated with or without inactivated EBV. The cells were then washed with cold PBS and resuspended in lysis buffer (Cell Signaling Technology, Inc., Danvers, MA, USA) and protease inhibitors (Roche Diagnostics GmbH, Mannheim, Germany), incubated on ice for 10 min, and centrifuged for 15 min at 15,000 rpm. The supernatant was then collected (whole-cell extract) and stored at −80 °C until use. To prepare the nuclear extract, precipitated cells were resuspended in cytoplasmic lysis buffer (Chemicon International, Temecula, CA, USA) and incubated for 15 min on ice. The cells were vortexed and then centrifuged for 10 min at 15,000 rpm, and the supernatant was removed. The cell pellets were washed twice with cytoplasmic buffer to remove any trace of proteins from the cytoplasmic extracts, resuspended in nuclear lysis buffer (Chemicon International, Temecula, CA, USA), and incubated on ice for 15 min. The cell suspensions were then sonicated for 10 s and centrifuged for 15 min at 15,000 rpm, and the supernatant fractions were stored at −80 °C. Pierce Microplate BCA Protein Assay Kit - Reducing Agent Compatible kits (Thermo Fisher Scientific, Inc., Waltham, MA, USA) were used to standardize the protein concentration in all samples.

### 4.6. Western Blot Analysis

The experimental procedures for western blot analysis were performed as previously described [38,39]. Briefly, equal amounts of the protein samples (20 μg) were separated using sodium dodecyl sulfate-polyacrylamide gel electrophoresis and transferred to a polyvinylidene fluoride membrane (EMD Millipore Corporation, Billerica, MA, USA). The membrane was probed with the appropriate antibodies, and immunoreactive proteins were visualized using SuperSignal West Pico enhanced chemiluminescence kits (Thermo Fisher Scientific, Inc.). Protein bands were detected using the ChemiDoc XRS System (Bio-Rad, Hercules, CA, USA).

### 4.7. Transfection and Luciferase Assay

HGFs (1 × 10^5^ cells/mL) were transfected with 20 ng of pGL3-κB luc using FuGENE 6 transfection reagent (Roche Diagnostics) according to the manufacturer’s protocol [38,39]. Subsequently, 24 h after transfection, the cells were treated with EBV for an additional 8 h. These cells were then harvested using Passive Lysis Buffer (Promega Corporation, Madison, WI, USA), and the extracts were assessed for luciferase activity using the Dual-Luciferase Assay System (Promega) as previously described [38,39]. All experiments were performed in triplicate, and the data are presented as the fold increase in luciferase activity (mean ± standard deviation (SD)) relative to controls.

### 4.8. Osteoclast Formation and TRAP Staining

RAW264.7 cells were seeded in 96-well culture plates (4 × 10^3^ cells/well). After culturing overnight, the medium was replaced with 200 μL of α-minimal essential medium containing 10% FBS and 50 ng/mL soluble RANKL (R&D Systems) in the absence or presence of the indicated copy numbers of EBV. The cultures were then incubated at 37 °C for 4 days. The medium was replaced every other day. Cells were fixed with 4% paraformaldehyde and stained for TRAP activity as described previously [40].

## Data Availability

The data presented in this study are available in the article.
